# Neuroendocrine Cell Carcinoma of Unknown Primary Arising in Long Standing History of Multiple Sclerosis

**DOI:** 10.1155/2015/135976

**Published:** 2015-03-24

**Authors:** Stergios Boussios, Vasiliki Kostadima, Anna Batistatou, Ioannis Tourkantonis, George Fotopoulos, Maria I. Argyropoulou, Nicholas Pavlidis

**Affiliations:** ^1^Department of Medical Oncology, Medical School, University of Ioannina, 45110 Ioannina, Greece; ^2^Department of Neurology, Medical School, University of Ioannina, 45110 Ioannina, Greece; ^3^Department of Pathology, Medical School, University of Ioannina, 45110 Ioannina, Greece; ^4^Department of Radiology, Medical School, University of Ioannina, 45110 Ioannina, Greece

## Abstract

Multiple sclerosis (MS) is a chronic autoimmune disease that targets myelinated axons in the central nervous system (CNS). Cancer of unknown primary site (CUP) is a well-recognised clinical disorder, accounting for 3–5% of all malignant epithelial tumors. CUP is clinically characterised as an aggressive disease with early dissemination. Studies of cancer risk in MS patients have shown inconsistent findings. An increased risk of malignancy in patients with MS has been suggested, but recently serious questions have been raised regarding this association. Use of disease-modifying therapies might contribute to an increased cancer risk in selected MS patients. The concurrence of MS and CUP is exceptionally rare. Here we describe the case of a neuroendocrine carcinoma of unknown primary diagnosed in a male patient with a nine-year history of MS. The discussion includes data from all available population-based register studies with estimates of certain malignancies in patients with MS.

## 1. Introduction

MS affects almost 1.3 million people worldwide [1]. Although the underlying cause of this entity is unknown and the etiology is not well understood, it is thought to be an autoimmune disease mediated by the degradation of myelin and destruction of axons in the CNS.

CUP is defined as histologically confirmed metastases in the absence of identifiable primary tumor despite a standardized diagnostic approach [2]. CUP accounts for 3–5% of all cancers, with a reported annual incidence per 100,000 population of 18 cases in Australia, 7–12 cases in the US, and six cases in Netherlands, ranking as the fourth commonest cause of cancer death in both sexes [3].

Here, we describe for the first time in the literature a case of neuroendocrine CUP which presented in a patient with long standing history of MS. Meanwhile, we review the literature in order to determine the underlying risk of cancer development in MS patients compared to the general population.

Since 2003, patients with CUP are distinguished into favourable and unfavourable groups. Favourable CUP group represents the 20% of the cases and experiences better responses to locoregional and/or systemic treatment and longer survival. Neuroendocrine CUP belongs to the favourable group [4].

## 2. Case Report

**Figure 1:**
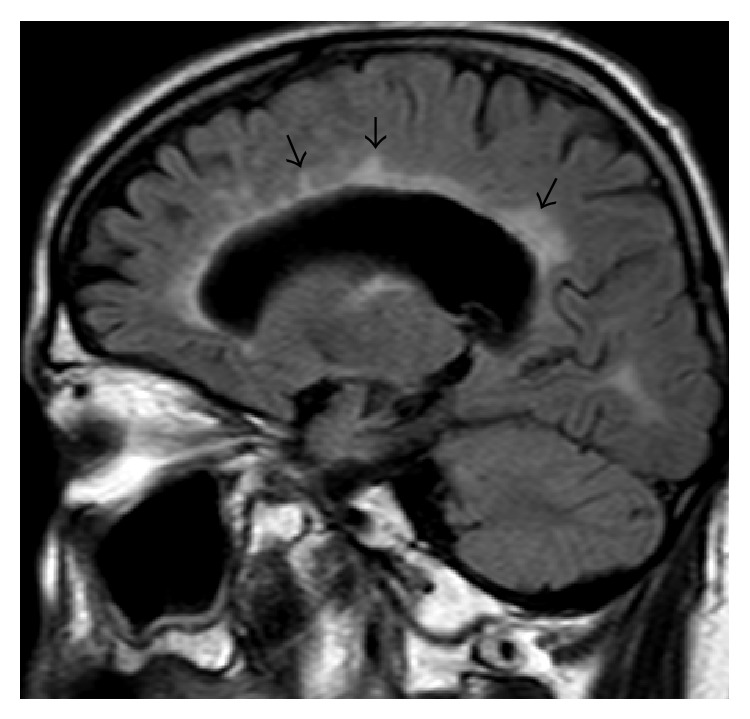
Left parasagittal FLAIR image of the brain of the patient at the time of his relapse (March 2012). Multiple bright white matter lesions with the long axis perpendicular to the surface of the lateral ventricle compatible with multiple sclerosis.

Our patient was first admitted to the neurology department of our hospital in October 2005 at the age of 52 years old because of disequilibrium. Preceding symptoms were also reported, including bilateral numbness of the lower legs and progressive weakness at the left leg attributed to hip replacement. As the patient was alcoholic, the initial explanation of the clinical picture was the polyneuropathy. At this time, a lumbar spine computed tomography (CT) scan was performed which disclosed a stenosis. Magnetic resonance imaging (MRI) of the brain revealed multiple lesions bright on T2-weighted images and enhancing after IV contrast administration. The cerebrospinal fluid (CSF) analysis detected an increased immunoglobulin G (IgG) or Link index (0.82) and positive CSF-specific oligoclonal bands by isoelectric focusing, without evidence of barrier injury. CSF tests were negative for virus isolation and for Gram stain and culture. A survey for vasculitis, including anti-nuclear antibody (ANA) and anti-phospholipid antibody, was negative too. During the following period, he experienced occasional relapses, mainly spinal and brainstem attacks with progression of his left leg motor weakness and incoordination in the upper limbs. He was treated for his relapsing-remitting disease with interferon beta-1a (IFN *β*-1a), beginning in 2007; treatment was switched in 2008 to IFN *β*-1b, because of a breakthrough spinal attack with increasing disability and less satisfactory response to steroids, with incomplete recovery. His disease then became more steadily progressive. In 2008, the patient underwent thoracic discectomy due to cervical spinal stenosis associated with hypertrophic degenerative changes. In March 2012, he presented with asymmetrical spastic paraparesis, generalized increased reflexes, spastic and ataxic gait, and moderate arm, leg, and truncal dysmetria. Neurologic examination revealed atrophy of intrinsic hand muscles. Gradual decline in cognition was also reported. Because of these new symptoms, the patient underwent brain MRI, which revealed multiple lesions involving supratentorial and infratentorial structures with cerebral and cerebellar atrophy on the T2-weighted images. Lesions of the periventricular white matter were perpendicular to the wall of the lateral ventricles (Figure 1). Normal results for blood testing and cancer workup were detected. After gadolinium contrast injection, ring enhancement over these lesions was noted on T1-weighted images. Thus, IFN-*β*-1b was discontinued and glatiramer acetate was begun which has been maintained without further deterioration.

**Figure 2:**
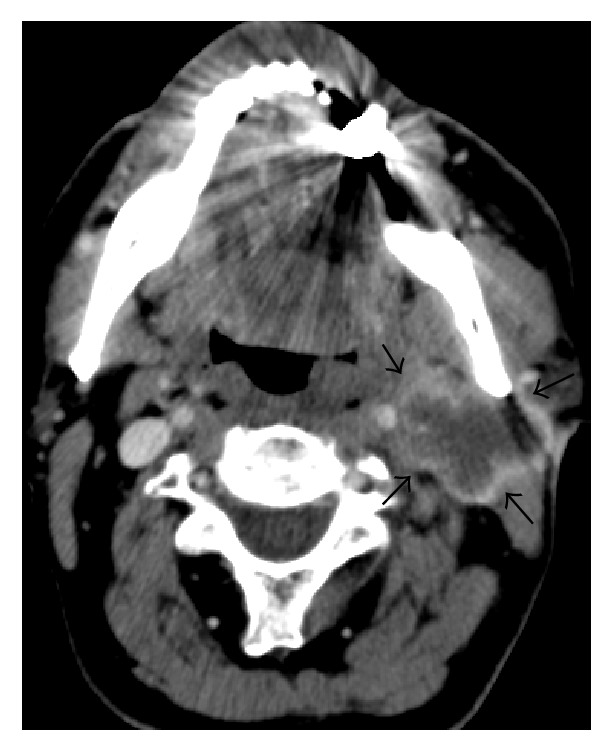
Axial postcontrast CT image of the neck showing left lymphadenopathy with necrotic center and peripheral enhancement.

**Figure 3:**
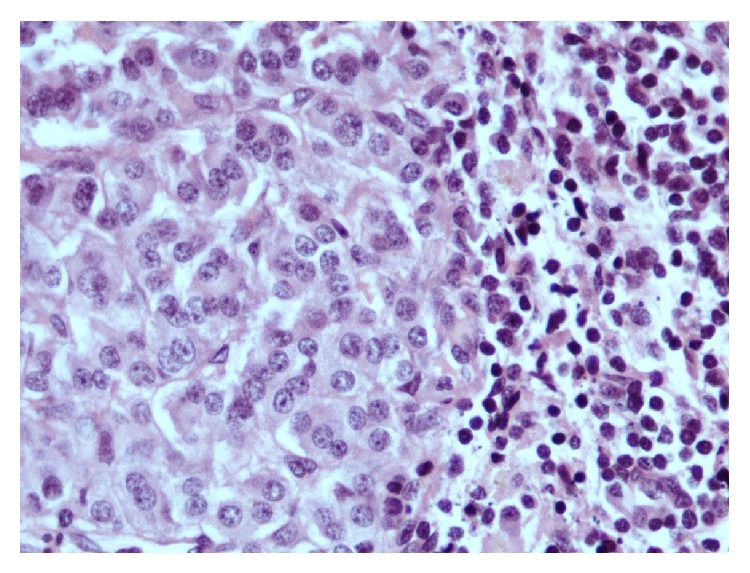
Infiltration of the lymph node by a metastatic neoplasm consisting of ovoid cells, with “salt and pepper” nuclei (haematoxylin-eosin stain ×400).

**Figure 4:**
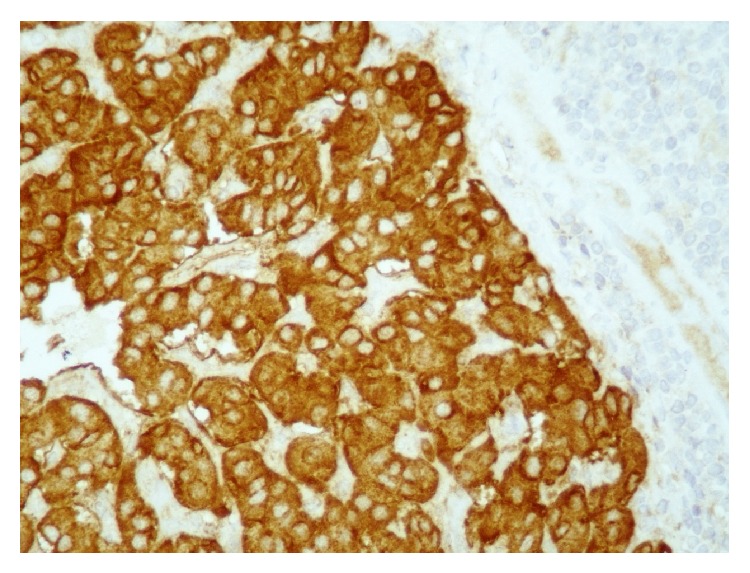
On immunohistochemical examination the cells stained positive for chromogranin (DAB ×400).

In May 2014, the patient presented with pain and swelling of the left side of his neck in association with a sore throat and chest tightness. The pain radiated to the left axilla and shoulder. There was no history of fever or night sweats. On examination, there was diffuse tender swelling on the left side of neck with a few scattered small tender lymph nodes in the posterior triangle of the left side of the neck. The remainder of clinical examination and the initial laboratory investigations were normal. Ultrasound of the neck demonstrated enlarged lymph nodes in the left supraclavicular fossa which were suspicious of malignancy. The patient underwent an excisional biopsy which revealed replacement of normal node architecture by metastatic neuroendocrine tumor (Figure 3). Positive immunohistochemical markers were neuron specific enolase, cytokeratin- (CK-) AE1/AE3, CK8/18, and chromogranin (Figure 4). The neuroendocrine features in the case of our patient were identified by immunohistochemical staining for chromogranin. However, the positivity was almost 20% which is moderate to strong staining intensity. These tumors have been termed “poorly differentiated neuroendocrine tumors” of unknown primary site. In order to find out the primary site the patient underwent panendoscopy of his upper aerodigestive tract which was normal. Further investigations failed to establish a possible primary site. This included proctosigmoidoscopy, tumor marker study, and CT scan of paranasal sinuses. A subsequent positron emission tomography also failed to identify a primary site malignancy and detected metastatic infiltration to the bilateral superior deep cervical nodes and axillary and right inguinal lymph nodes. No other lesions were demonstrated, with the liver and lungs in particular appearing normal. A diagnosis of metastatic neuroendocrine tumor of unknown primary site was eventually made and the patient was referred to the oncology department. He received palliative radiation to cervical and supraclavicular lesions to relieve local symptoms and he achieved a partial response. No chemotherapy was attempted at that time in order to avoid additional toxicity. After 10 months of follow up he complained of a palpable mass in his left neck. A CT scan of the neck demonstrated the presence a mass lesion with central necrosis and peripheral enhancement compatible with local relapse (Figure 2). As the favorable risk subsets should be treated similarly to patients with equivalent known primary tumors with metastatic dissemination, a platinum-based combination schedule was attempted. Although a partial clinical response was detected after two cycles, the patient stopped chemotherapy due to grade IV myelotoxicity and to exacerbation of his neurological syndrome.

## 3. Discussion

Concurrence of CUP and MS has not been reported in the literature. Here, we describe an intriguing case of overlap between these two entities.

Multiple studies have examined the relationship between MS and cancer risk. The majority of these analyses have concluded that there is no difference in the risk of developing malignancies between patients with MS and the general population. However, some studies have suggested either increased risk in breast, brain, and bladder tumors, in patients with MS, or reduced risk in cancers of the digestive and respiratory tract.

A small but significantly increased risk of breast cancer in patients with MS was reported in a few studies. Although the Danish MS Registry (DMSR) study failed to show an increased overall cancer risk in women with MS (SIR, 1.01), there was an increased risk of breast cancer (SIR, 1.21) [5]. The study also revealed that patients with MS tended to have larger tumors at diagnosis compared with patients without MS. The etiology of the increased breast cancer risk in MS patients is currently obscure and deserves further investigation.

Reports of MS mimicking brain tumors clinically and radiologically could potentially explain the reverse association of brain cancer before MS [6, 7], as could a brain tumor associated with T2 hyperintensity on MRI mimicking MS. In the British Columbia MS cohort, the nonsignificant elevated risk for brain cancer was most evident in a short time after disease onset. The diagnostic workup of MS may provide an explanation (i.e., surveillance bias) and is in accordance with other reports that have observed an increased risk [8–11]. Other studies have found no significant increase in brain cancer risk [5, 12–14].

In the DMSR study, the risk of bladder cancer overall was not statistically significantly increased [SIR = 1.19 (0.93–1.51), *n* = 66] [5]. Moreover, female MS patients demonstrated an increasing risk of bladder cancer in excess of 10 years after MS onset [SIR = 1.58 (1.04–2.40), *n* = 22]. On the other hand, the risk of bladder cancer in men was increased 1 to 9 years after MS diagnosis [SIR = 2.09 (1.23–3.52), *n* = 14] [5]. Approximately, the same results were obtained in the British Columbia MS cohort concerning the risk of developing bladder cancer (SIR: 1.21; 95% CI: 0.73–1.88) [8].

The reduced risk of digestive tract cancers in British Columbia cohort is in accordance with findings from the Oxford record linkage study (ORLS) [10], studies from Scandinavia [12], France [14], and a US veterans study [15], although not always statistically significant. Several epidemiologic studies have suggested that long-term users of nonsteroidal anti-inflammatory drugs (NSAIDs) have a lower risk of colorectal cancers compared to nonusers [16–19]. Whether the reduced cancer risk of digestive organs in MS patients in a true phenomenon or due to lifestyle factors or medications was used remains to be elucidated.

A total of five studies, reporting measures of the risk of lung cancer for an MS cohort relative to population controls, were studied [5, 9, 10, 12, 14]. The heterogeneity between the studies was not significant (*P* = 0.87). Interestingly enough, it has reported a significantly decreased risk of lung cancer in the MS cohort relative to controls [OR 0.67 (95% CI 0.59–0.76) *P* < 0.00001] [20]. A possible explanation of the decreased lung cancer prevalence would be the increased immune surveillance in MS. The considerable reduction in lung cancer in MS patients despite a predicted increase in risk provides assurance with intensive in vitro and in vivo study of mechanisms underlying this point.

Since 1993 several disease-modifying agents were introduced and proven to alter the natural history of MS, including IFN-*β*1b, IFN-*β*1a, and glatiramer acetate. IFN-*β* blocks the potent proinflammatory agent IFN-*γ* and other inflammatory cytokines that can cause immune-mediated damage to myelin cells [21–23]. Glatiramer acetate is a synthetic polymer of amino acids that was designed to mimic myelin basic protein and act as a decoy to lessen autoimmune-mediated damage to actual myelin cells [24]. Newer research has shown that the drug is involved in a TH1-TH2 shift and subsequent increased secretion of anti-inflammatory cytokines [25].

## 4. Conclusions

The majority of the evidence currently suggests that patients with MS are not at an overall increased risk for the development of cancer, apart from a possibility of breast, bladder, and brain neoplasms. In addition, there is some evidence for a reduced cancer risk for digestive tract organs and respiratory organs.

We assume that our unusual report, in addition to the literature on the concurrence of cancer and MS, might be useful to stimulate further analyses about the serious questions that have been raised regarding this association.
